# Mathematical morphology-based approach to the enhancement of morphological features in medical images

**DOI:** 10.1186/2043-9113-1-33

**Published:** 2011-12-16

**Authors:** Yoshitaka Kimori

**Affiliations:** 1 Imaging Science Division, Center for Novel Science Initiatives, National Institutes of Natural Sciences, Toranomon 4-3-13, Minato-ku, Tokyo, 105-0001, Japan

**Keywords:** Mathematical morphology, Contrast enhancement, Mammographic image, Chest radiographic image, Retinal image

## Abstract

**Background:**

Medical image processing is essential in many fields of medical research and clinical practice because it greatly facilitates early and accurate detection and diagnosis of diseases. In particular, contrast enhancement is important for optimal image quality and visibility. This paper proposes a new image processing method for enhancing morphological features of masses and other abnormalities in medical images.

**Method:**

The proposed method involves two steps: (1) selective extraction of target features by mathematical morphology and (2) enhancement of the extracted features by two contrast modification techniques.

**Results:**

The goal of the proposed method is to enable enhancement of fine morphological features of a lesion region with high suppression of surrounding tissues. The effectiveness of the method was evaluated in quantitative terms of the contrast improvement ratio. The results clearly show that the method outperforms five conventional contrast enhancement methods. The effectiveness and usefulness of the proposed method were further demonstrated by application to three types of medical images: a mammographic image, a chest radiographic image, and a retinal image.

**Conclusion:**

The proposed method enables specific extraction and enhancement of mass lesions, which is essential for clinical diagnosis based on medical image analysis. Thus, the method can be expected to achieve automatic recognition of lesion location and quantitative analysis of legion morphology.

## Background

In contemporary medical practices, image-based diagnosis is a crucial component of disease evaluation. Medical images of various modalities such as X-ray, mammography, computed tomography, magnetic resonance imaging, color fundus imaging, and ultrasound contain important information for clinical diagnosis.

**Figure 1:**
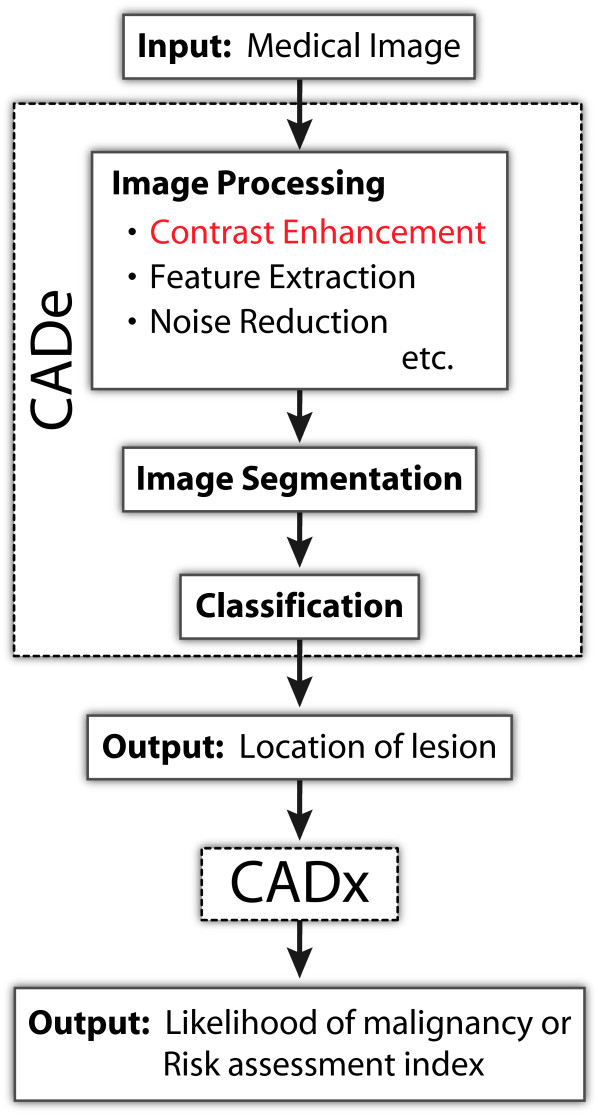
**Schematic representation of typical CAD system**.

Computer-aided detection (CADe) and/or diagnosis (CADx) [1-4] is used to assist physicians in interpreting image-based information accurately and efficiently. CADe is the system of identifying the location of potential lesions within a medical image. CADx is the system of evaluating or characterizing the lesions, which were initially located by CADe. In general, both systems are referred to collectively as CAD. It includes the following fundamental components: image processing, image segmentation, classification, registration, modeling, visualization, etc. The schematic diagram of typical CAD system is shown in Figure 1.

Of these, image processing is of particular importance. Its goals are threefold: to improve original image data, extract morphological features of pathological structures, and obtain relevant information essential for clinical diagnosis. Image processing is also indispensable for subsequent preprocesses such as image segmentation and classification in CADe system.

This study focuses on contrast enhancement in image processing. Medical images are often characterized by low grey-level contrast and complicated structured backgrounds. In processing of such images, contrast enhancement involves highlighting small diagnostic features that are superimposed on a complex background. Various contrast enhancement techniques have been proposed [5-7], including histogram modification methods, spatial domain and frequency domain filtering, and mathematical morphology-based methods. Let us consider these in order.

*Histogram modification *is popular because of its simplicity, speed, and capability to preserve all information from the original image. Typical histogram modification techniques include histogram equalization and its variants [8-10].

*Spatial domain filtering *involves direct manipulation of pixel values on an original image plane [11]. Spatial domain filtering is often implemented with a convolution mask, which is a small matrix of fixed numbers [12-14].

*Frequency domain filtering *involves transformation of an original image into the frequency domain by means of discrete Fourier transform, discrete cosine transform, or discrete wavelet transform [15,16].

A common disadvantage of these methods is that they usually enhance entire structures in a medical image without discrimination, whereas, for effective detection of lesions, it is necessary to enhance only specific target lesions and not the surrounding tissue. For this purpose, the author attempted to devise a new image processing method based on *mathematical morphology*. This is a nonlinear image analysis method based on the set theory and involves extraction of shape characteristics from an image, typically for shape representation and description [17]. Furthermore, size information in the image can also be obtained by using the granulometry which is an approach to measure a size distribution of objects [18]. Several morphological contrast enhancement methods have been applied to medical images [19-22]. These methods enable detection of lesions of various sizes and shapes, including complex shapes.

This study describes an approach to medical image processing using a type of mathematical morphology called *rotational morphological processing *(RMP) [23-25] for enhancement of mass lesions in medical images captured by various modalities. The proposed method involves two consecutive steps: (1) selective extraction of target features by mathematical morphology and (2) enhancement of the extracted features by two contrast modification techniques. This method enables specific enhancement of target lesion features with high suppression of complex background tissues.

The method was evaluated subjectively and quantitatively in terms of the measured contrast improvement ratio (*CIR*) in a simulated mammographic image. It was then applied to three real medical images: a mammographic image, a chest radiographic image, and a retinal image. The results show that the method is highly efficient and reliable.

This paper is organized as follows: the current section presents relevant background information. The methods section describes the sample images and methodology used in the study. The results and discussion section describes the experimental results. The final section presents the study conclusions.

## Methods

### Sample images

Three types of medical images were used to test the performance of the proposed method: mammographic, chest radiographic, and retinal.

Mammographic images were obtained from the mini mammography database provided by the Mammographic Image Analysis Society (MIAS) [26]. The size of each mammographic image was 1024 × 1024 pixels with a spatial resolution of 200 μm/pixel.

Chest radiographic images were obtained from the standard digital image database for chest lung nodules and non-nodules provided by the Japanese Society of Radiological Technology [27]. Image size was 2048 × 2048 pixels with a spatial resolution of 0.175 mm/pixel.

Retinal images were obtained from the digital retinal images for vessel extraction (DRIVE) database provided by the Image Sciences Institute [28]. Image size was 584 × 565 pixels.

### Image processing using mathematical morphology

#### Types of mathematical morphology

Mathematical morphology [17] is a methodology for extracting shape and size information from an image. It involves configuration of a set of nonlinear operators that act on images by using structuring elements. The two basic morphological operators are dilation and erosion, from which many operations can be derived. For grey-scale image *f *and structuring element *B*, dilation and erosion are defined as follows:

(1) $$\text{dilation}:\delta_{B}{\left(f\right)}_{\left(x,y\right)}=\max\left\{f\left(x-s,y-t\right)+B\left(s,t\right)|\left(x-s\right),\left(y-t\right)\in D_{f};\left(s,t\right)\in D_{B}\right\},$$

(2) $$\text{erosion}:\varepsilon_{B}{\left(f\right)}_{\left(x,y\right)}=\min\left\{f\left(x+s,y+t\right)-B\left(s,t\right)|\left(x+s\right),\left(y+t\right)\in D_{f};\left(s,t\right)\in D_{B}\right\},$$

where for image *f *and structuring element *B*, (*x*, *y*) and (*s*, *t*) are the respective co-ordinate sets and *D_f _*and *D_B _*are the respective domains. For simultaneous use of dilation and erosion, opening and closing operations are derived as follows:

(3) $$\text{opening}:\gamma_{B}\left(f\right)=\delta_{B}\left(\varepsilon_{B}\left(f\right)\right),$$

(4) $$\text{closing}:\phi_{B}\left(f\right)=\varepsilon_{B}\left(\delta_{B}\left(f\right)\right).$$

In conventional morphological operations, a single structuring element is used to process an image. An operation involves passing a structuring element over an image and retaining, for further image processing, any features in the image that are fit by the structuring element. However, a structuring element can fit only same-direction features, not different-direction features. Thus, such operations are not suitable for use on randomly oriented features in an image.

To overcome this limitation, a type of extended mathematical morphology called RMP has been proposed [23-25]. RMP rotates the original image with respect to the structuring element that is fixed in one direction.

We assume that full 180° angles are equally divided into *N *directions; the function *f_i _*denotes the rotation of an original image *f *by the degree of *θ_i _*= 180 *i*/*N*, where *i *= 0, 1,..., *N*-1. The image *f_i _*is rotated clockwise on the centre of the image frame. The opening operation of the rotated image *f_i _*with *B *is represented as *ϕ_B_*(*f_i_*), and the closing operation of the rotated image *f_i _*with *B *is represented as *γ_B_*(*f_i_*). The images operated by the RMP are followed by rotation at *θ_i _*degrees in the counter-clockwise direction. The *i-*th rotated opened image and closed image are denoted by *h_i_^Opn ^*and *h_i_^Clsn^*, respectively. The processed images are finally compiled together by combining pixel values as per certain rules. The maximum brightness value of each pixel is selected in opening processing and the minimum brightness of each pixel value is selected in closing processing.

Opening and closing by the RMP are denoted as *ϕ'_B_*(*f *) and *γ'_B_*(*f *), respectively. These are defined as follows:

(5) $$\text{opening by RMP}:\gamma_{B}^{\prime }{\left(f\right)}_{\left(x,y\right)}\;=\;\underset{i\in \left(0,1,\ldots ,N-1\right)}{\max}\;\left\{\;h_{i}^{Opn}\left(x,y\right)\;\right\},$$

(6) $$\text{closing by RMP}:\phi_{B}^{\prime }{\left(f\right)}_{\left(x,y\right)}\;=\;\underset{i\in \left(0,1,\ldots N-1\right)}{\min}\;\left\{\;h_{i}^{Clsn}\left(x,y\right)\;\right\}.$$

Furthermore, two filters now defined: a smoothing filter *SM*(*f *) and feature extraction filter δ^*TH*^(*f *). Smoothing filter *SM*(*f *) is a redefinition of the LOCO filter [29], which uses the opening and closing operations, and is defined as follows:

(7) $$SM\;\left(f\right)=\frac{1}{2}\;\left\{\;\gamma_{B}^{\prime }\left(\phi_{B}^{\prime }\left(f\right)\right)\;\right\}\;+\;\frac{1}{2}\;\left\{\;\phi_{B}^{\prime }\left(\gamma_{B}^{\prime }\left(f\right)\right)\;\right\}.$$

Feature extraction filter δ^*TH*^(*f *) calculates the difference between original image *f *and its smoothed image *SM*(*f *) and is defined as follows:

(8) $$\delta^{TH}\left(f\right)=f-SM\left(f\right).$$

The two filters operate in the following manner: Smoothing filter *SM*(*f *) creates a smoothed image by removing structures in the original image that are represented as bright and dark values. Feature extraction filter δ^*TH*^(*f *) recreates the original image by recovering these structures.

#### Structuring element selection

Two parameters of a structuring element, size and shape, determine the effect and performance of the morphological filter.

The size parameter of a structuring element must be set in accordance with the size of the structure to be extracted. The feature extraction filter extracts morphological features whose base sizes are smaller than the size of the structuring element. Hence, the size of the structuring element must be larger than the base size of the target lesion.

The shape parameter of a structuring element must be set in accordance with the shape of the structure to be extracted. For extraction of mass structures, a structuring element in the shape of a line segment (width 1 pixel) was chosen. This element shape was used in RMP for extraction of bright spots in fluorescence microscopy images, in which local spots with base diameters smaller than the length of the structuring element were extracted, confirming the effectiveness of morphological processing [24]. For extraction of fibrous (or elongated) structures, that is, spiculated masses, in mammographic images and for extraction of blood vessels in retinal images, disk-shaped structuring elements were chosen.

Thus, the proposed method should be capable of handling various morphological features by changing the size and shape of the structuring element.

#### Contrast enhancement method

The proposed method involves two steps: selective extraction of the features to be enhanced by feature extraction filter δ^*TH*^(*f *), followed by contrast enhancement of the extracted features by two contrast modification techniques.

**Figure 2:**
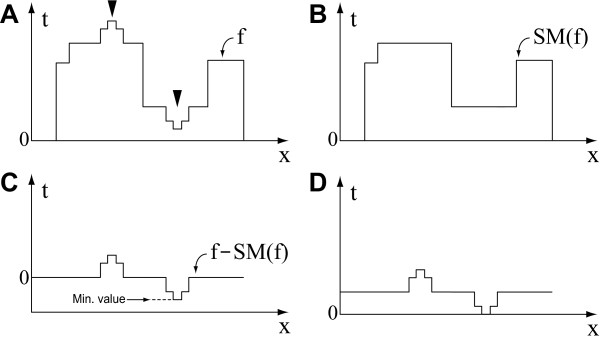
**Selective extraction of features**. (A) Original image *f*; target regions to be extracted are indicated by arrowheads. (B) Smoothing by *SM*(*f *). (C) Feature extraction by δ^*TH*^(*f *). (D) Representation of the integer range of the signal shown in C; the entire signal range is shifted up to the integer range and then the minimum value is set to zero in the new range.

Figure 2 shows the first step, selective extraction of features. Figure 2(A) shows the intensity profile of the original image one-dimensional signal *f*, where the positive and negative peaks (indicated by arrowheads) are the targets to be extracted. The signal is smoothed by *SM*(*f *) (Figure 2(B)), then filtered by δ^*TH*^(*f *) (Figure 2(C)). The resulting signal has negative values; hence, the value range is shifted and the minimum value is set to zero in the new range (Figure 2(D)).

In the second step, the contrast of the feature image is enhanced by application of the histogram equalization (HE) technique [11] followed by the linear contrast stretching (LCS) technique [11]. The former technique manipulates the histogram of an image by redistributing the number of pixels between intensity levels to obtain an equal frequency. The latter technique normalizes the intensity levels by determining the minimum and maximum intensity values of an image and then stretching this range linearly so that the full range is available for output intensity values (for example, 0-255 for an 8-bit grey-scale image).

#### Contrast improvement ratio (*CIR*)

The effectiveness of image enhancement was evaluated by measuring the *CIR *[30]. It is defined as the ratio of the enhanced and unenhanced images within region of interest (ROI) *R*, given as

(9) $$CIR=\frac{\underset{\left(x,y\in R\right)}{\sum }{\left|C\left(x,y\right)-\bar{C}\left(x,y\right)\right|}^{2}}{\Sigma_{\left(x,y\in R\right)}C{\left(x,y\right)}^{2}},$$

where *C *(*x, y*) and $\bar{C}(x,y)$ are the local contrast values at (*x, y*) of the unenhanced (original) and enhanced images, respectively. Local contrast value *C *(*x, y*) can be computed as

(10) $$C\left(x,y\right)=\frac{\left|p-a\right|}{p+a},$$

where *p *and *a *are the mean values within the center region (3 × 3) pixels and the neighborhood, or surrounding region, (7 × 7) pixels, respectively.

## Results and discussion

### Evaluation of contrast enhancement

For evaluation of contrast improvement, the proposed method was compared with five conventional contrast enhancement methods: HE, LCS, unsharp masking (USM) [11], multiscale retinex (MSR) [31], and contrast limited adaptive histogram equalization (CLAHE) [32].

**Figure 3:**
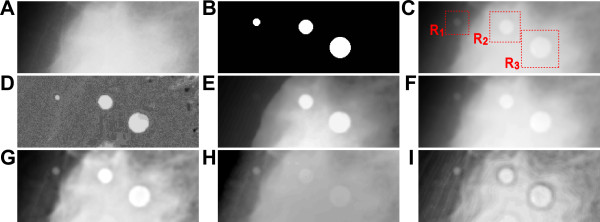
**Image enhancement achieved by various contrast enhancement methods**. (A) Subimage of mammographic image *mdb003*. (B) Phantom features of well-circumscribed masses *m*_1_, *m*_2_, and *m*_3 _of different diameters. (C) Simulated image; ROIs that include each phantom feature are denoted as *R*_1_, *R*_2_, and *R*_3_. (D-I) Contrast enhanced images obtained by the following methods: the proposed method (D), histogram equalization (E), linear contrast stretching (F), unsharp masking (G), multiscale retinex (H), and contrast limited adaptive histogram equalization (I).

Figure 3 shows the image enhancement achieved by the proposed and several conventional methods. The test image (Figure 3(C)) consists of a mammographic image (Figure 3(A)) superimposed with phantom features from a well-circumscribed mass (Figure 3(B)). A subimage (265 × 105 pixels) of the original image is shown. Phantom features *m*_1_, *m*_2_, and *m*_3 _are 11, 21, and 31 pixels (2.2, 4.2, and 6.2 mm) in diameter, respectively. The phantom features were smoothed by Gaussian filtering and then blended into the mammographic image. The areas bounded by dotted lines in Figure 3(C) (*R_1_*, *R_2_*, and *R_3_*, 35 × 35, 45 × 45, and 55 × 55 pixels, respectively) denote the ROIs for each phantom feature used for computing the *CIR*.

The simulated image was enhanced by the proposed method (Figure 3(D)) and by HE, LCS, USM, MSR, and CLAHE (Figures 3(E)-(I)). Feature extraction filter δ^*TH*^(*f *) was operated with a line segment structuring element (length 41 pixels or 8.2 mm) whose diameter is larger than the base diameter of each phantom feature. Relevant parameter settings were optimized for fair comparison as follows. USM involves creating a blurred version of the image using a Gaussian blur filter and then subtracting this from the original image using a weighted factor that controls the degree of enhancement. Accordingly, the blur radius of the Gaussian was set to 10 pixels and the mask weight was set to 0.7. MSR involves use of the typical retinex theory for image contrast enhancement and calculation of output image values by taking the difference between the original image and its blurred version in the logarithm domain. Accordingly, three convolution scales were used with standard deviations set to 5, 50, and 150 pixels, respectively, and weighted factors were set to 1/3 for each scale. CLAHE is an adaptive image contrast enhancement technique based on histogram modification, and it operates on small regions (blocks) in an image and improves the local contrast of the image. Accordingly, block size was set to 15 × 15 pixels.

Table 1 lists the measured *CIR *values for ROIs that include phantom features. For all phantom features, the proposed method achieves significantly higher *CIR *values-that is, remarkably higher contrast, and hence better results-than the other methods.

**Table 1:** Measured *CIR *values for ROIs including phantom features

ROI	Measured *CIR *values for various image enhancement methods					
	
	Proposed	HE	LCS	USM	MSR	CLAHE
*R_1_*	14.8291	1.3832	0.0337	2.1091	0.5043	3.8390

*R_2_*	199.3442	9.8188	0.0330	1.9410	0.5647	11.7008

*R_3_*	229.1748	8.3543	0.0903	2.0105	0.3285	12.6779

Proposed method, histogram equalization (HE), linear contrast stretching (LCS), unsharp masking (USM), multiscale retinex (MSR), and contrast limited adaptive histogram equalization (CLAHE).

The local contrast value (Eqn. (10)) is the difference in average grey-scale values between a target feature and the surrounding tissue. For enhancement by the proposed method (Figure 3(D)), inhomogeneous background is subtracted and each phantom feature is clearly isolated in the ROIs. Thus, the luminance of the phantom features is high and, in contrast, the luminance of the background is low. Increase in local contrast may contribute to the high *CIR *value.

The most distinctive property of the proposed method is that it enhances only the target features. This property improves the visibility of subtle mass lesions and reduces unwanted background information even in real medical images.

### Application results

**Figure 4:**
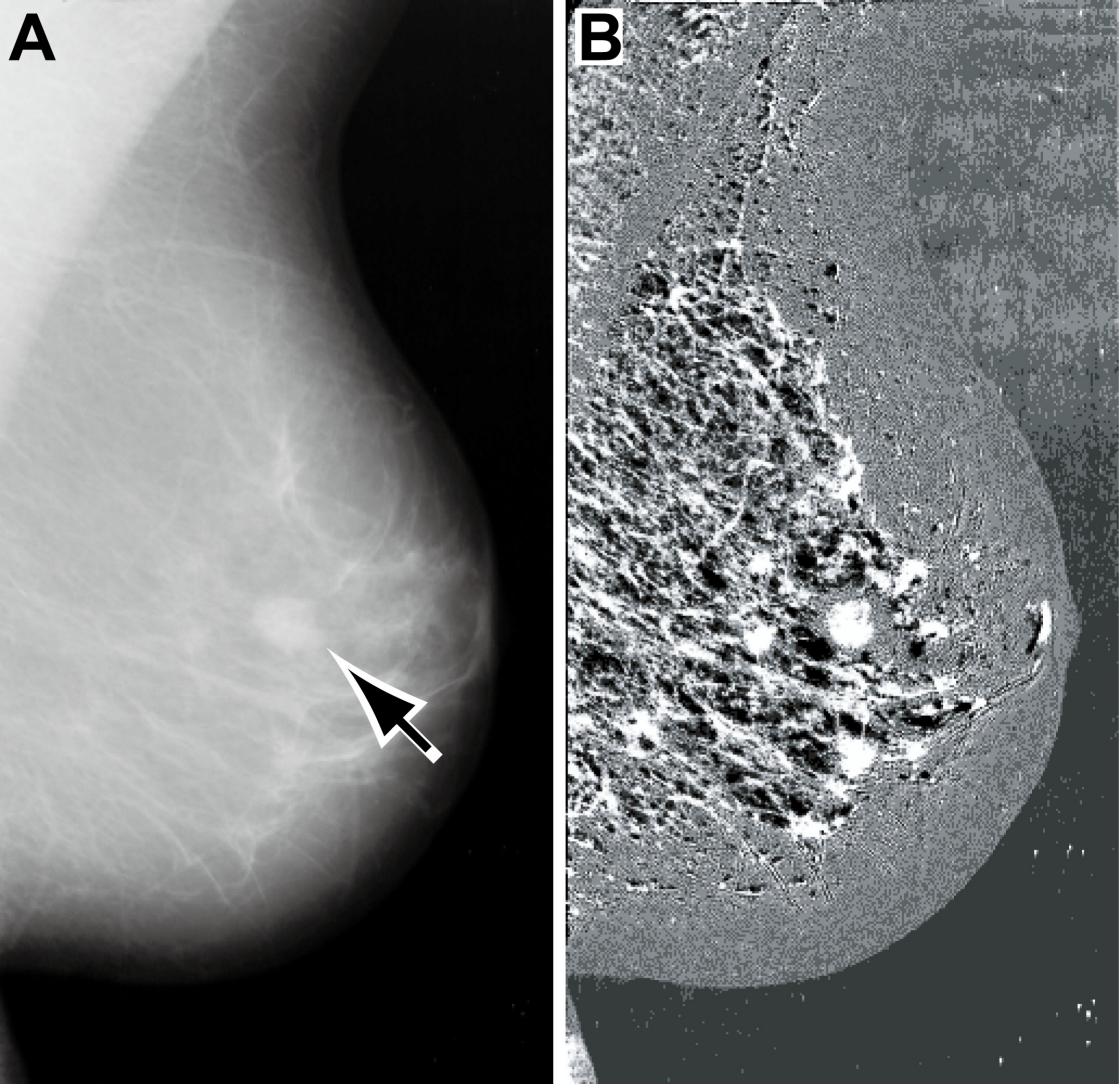
**Image enhancement achieved by the proposed method for a mass lesion in a mammographic image**. (A) Original mammographic image (*mdb010*); the location of the mass lesion is indicated by the arrow. (B) Enhanced image obtained by the proposed method.

Figure 4 shows the image enhancement achieved by the proposed method for a mammographic image containing a mass lesion. In the original image (Figure 4(A), resized to half its original size, resolution 400 μm/pixel), the arrow points to the mass lesion. The enhanced image (Figure 4(B)) shows the lesion extracted with a line segment structuring element (length 31 pixels or 12.4 mm). As observed from the figure, the target mass lesion is isolated, other suspicious regions containing abnormalities in surround tissues are more clearly evident, contrast throughout the entire breast region is improved, and the breast border contour can be identified easily. Information about the breast boundary is used in several mammogram registration or mammogram segmentation techniques that are keys to improved automatic clinical diagnosis [33,34]. It is hoped that the proposed method will prove useful in these advanced techniques.

**Figure 5:**
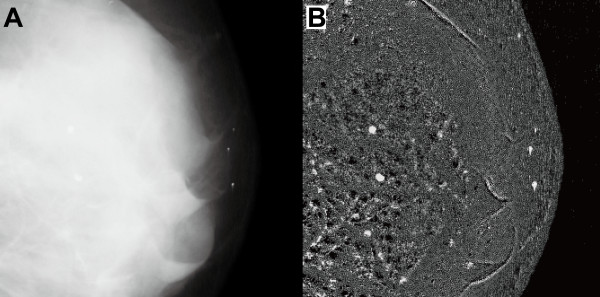
**Image enhancement achieved by the proposed method for calcifications in a mammographic image**. (A) Subimage of original mammographic image (*mdb002*). (B) Enhanced image obtained by the proposed method.

Figure 5 shows the image enhancement achieved by the proposed method for a mammographic image containing suspicious calcifications. In the original image (Figure 5(A), 331 × 331 pixels, resolution 200 μm/pixel), the calcifications are not clearly visible. The enhanced image (Figure 5(B)) shows the calcifications extracted with a line segment structuring element (length 11 pixels or 2.2 mm). It is observed that contrast enhancement clearly improves the visibility of the calcification shapes and boundaries, which can now be discriminated clearly from inhomogeneous background.

**Figure 6:**
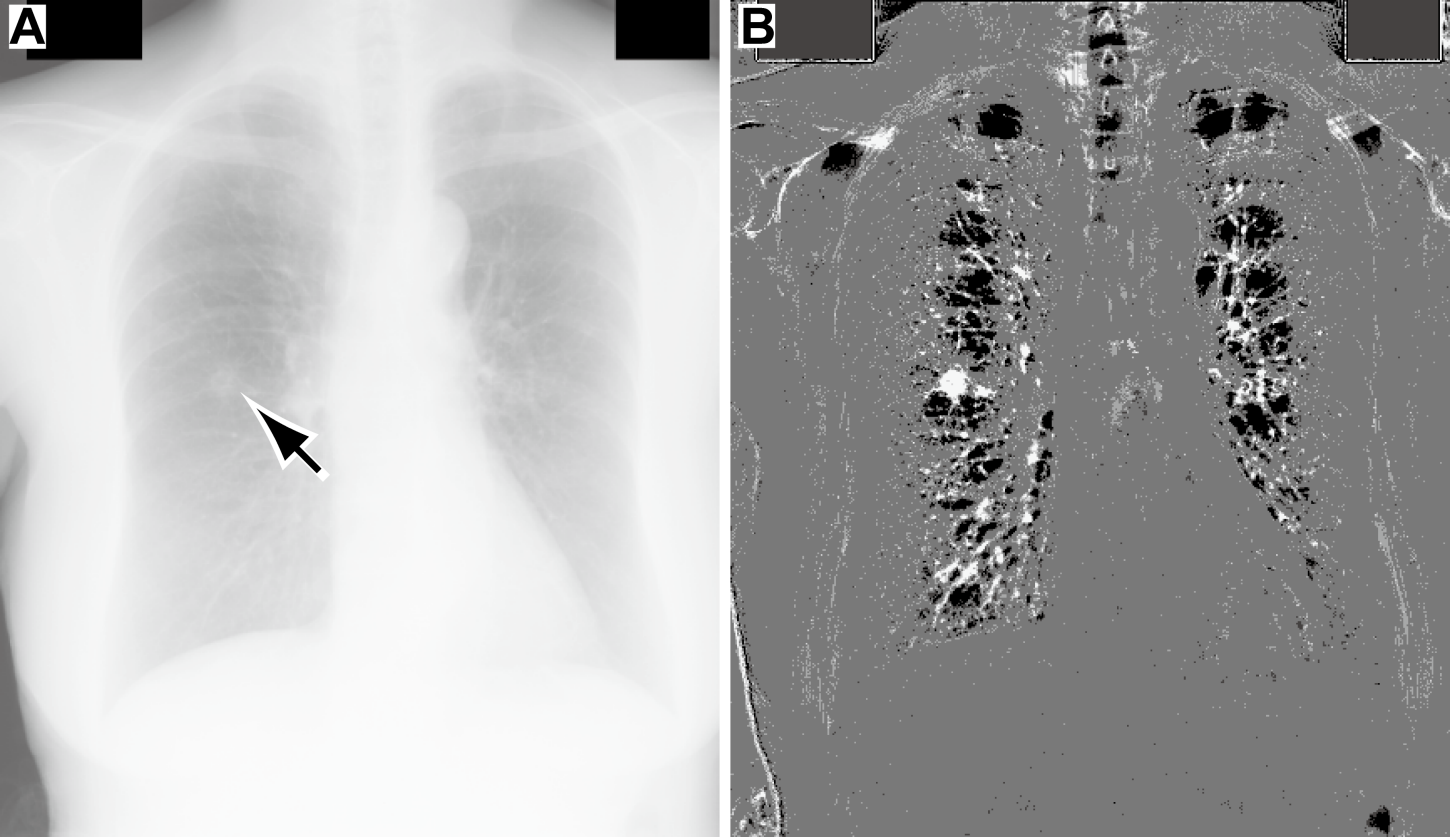
**Image enhancement achieved by the proposed method for a nodule in a chest radiographic image**. (A) Original chest radiographic image (*JPCLN044*); the location of the single lesion (pulmonary nodule) is indicated by the arrow. (B) Enhanced image obtained by the proposed method.

Figure 6 shows the image enhancement achieved by the proposed method for a chest radiographic image (*JPCLN044*). In the original image (Figure 6(A), resized to 512 × 512 pixels, resolution 0.7 mm/pixel), the arrow points to the single lesion (pulmonary nodule). The nodule overlaps with a rib in the lung field. The enhanced image (Figure 6(B)) shows the nodule extracted with a line segment structuring element (length 31 pixels or 21.7 mm). The figure shows that the nodule is more clearly evident and the ribs and the surrounding lung parenchyma are suppressed.

**Figure 7:**
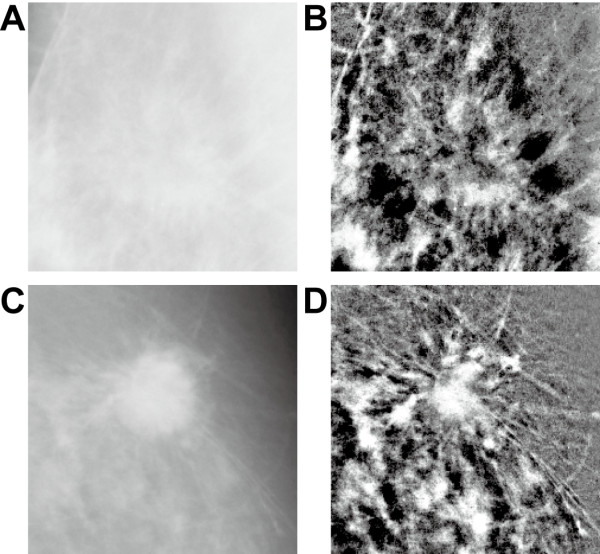
**Image enhancement achieved by the proposed method for a spiculated margin**. (A, C) Subimages of original mammographic images (*mdb191 *and *mbd202*, respectively). (B, D) Enhanced images obtained by the proposed method.

Figure 7 shows the image enhancement achieved by the proposed method for a spiculated margin in mammography. Figures 7(A) and 7(C) show subimages (227 × 227 pixels, resolution 200 μm/pixel) extracted from *mdb191 *and *mbd202*, respectively. Figures 7(B) and 7(D) show the margin enhanced with a disk-shaped structuring element (diameter 41 pixels or 8.2 mm). Local contrast is significantly improved, and morphological details of the speculated margins whose widths are smaller than the diameter of the structuring element are clearly visible. For radiologists, the shape of the mass margin is the most important diagnostic factor for evaluating the degree of malignancy/benignancy of a lesion. Thus the proposed method should aid greatly in correct diagnosis.

**Figure 8:**
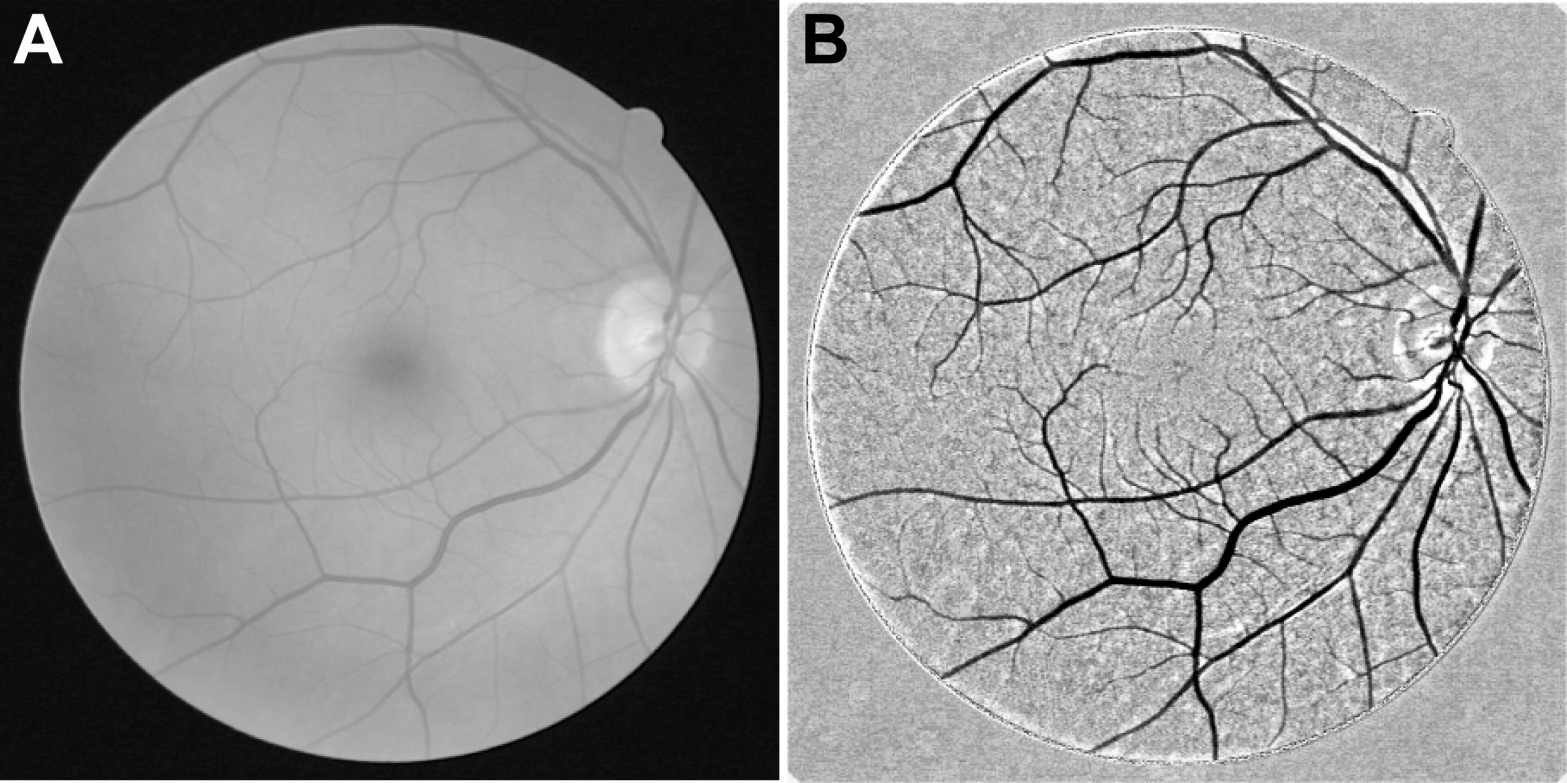
**Image enhancement achieved by the proposed method for vessels in a retinal image**. (A) Original retinal image (*16_test*). (B) Enhanced image obtained by the proposed method.

Figure 8 shows the image enhancement achieved by the proposed method for blood vessels in a retinal image. In the original image (Figure 8(A), 565 × 584 pixels), vessel width varies from 1 to 10 pixels. The enhanced image (Figure 8(B)) shows the vessels extracted with a disk-shaped structuring element (diameter 11 pixels). Local contrast is clearly improved, and now even narrow vessels are visible. Although vessels are represented as dark features against a bright background, they are extracted independently of their luminance because extraction by the proposed method is based on the shape of the target feature.

When the original image is noisy, noise reduction is necessary as a preprocessing for contrast enhancement. As a more straightforward approach, a blurring (or smoothing) filter can be used to this purpose. In general, contrast of the original image further decreases with reducing noise by the blurring filter. However, it is considered that the proposed method can be applied to such degraded images. As a future work, it is necessary to develop the method for discriminating between signal and noise in many practical medical images.

## Conclusion

Image contrast enhancement is important for medical diagnosis, and early detection of disease is facilitated by specific enhancement of low-contrast lesion features. For contrast enhancement of medical images, this paper presents a new method based on RMP. The method involves two steps: selective extraction of target features followed by enhancement of the extracted features. It offers the following advantages:

• It can be applied to various types of medical images without restrictions.

• It enables specific enhancement of target lesion features with high suppression of complex background tissues.

• It can handle various morphological features by changing the size and shape of the structuring element.

• It enables enhancement of both bright and dark morphological features.

The superior performance of the proposed method was demonstrated by comparison of *CIR *values with various conventional methods, and the effectiveness and usefulness of the method were demonstrated by application to various types of medical images.

Future studies should involve development of an automatic image segmentation method to separate targets of interest from their backgrounds. This method is important for automatic recognition of abnormalities and the quantitative analysis of medical images using CAD. The proposed method, which successfully distinguishes between targets and surrounding tissue, may, in combination with conventional automatic thresholding techniques, enable the development of a new approach for automatic segmentation of medical images.

## Competing interests

The author declares that they have no competing interests.

## Authors' contributions

YK conceived the study, developed the algorithms, implemented the image processing/analysis, and drafted the manuscript.
